# Linking seasonal home range size with habitat selection and movement in a mountain ungulate

**DOI:** 10.1186/s40462-017-0119-8

**Published:** 2018-01-05

**Authors:** Duarte S. Viana, José Enrique Granados, Paulino Fandos, Jesús M. Pérez, Francisco Javier Cano-Manuel, Daniel Burón, Guillermo Fandos, María Ángeles Párraga Aguado, Jordi Figuerola, Ramón C. Soriguer

**Affiliations:** 1 Estación Biológica de Doñana, CSIC, C/Américo Vespucio, s/n, E-41092 Sevilla, Spain; 2 German Centre for Integrative Biodiversity Research (iDiv) Halle-Jena-Leipzig, Deutscher Platz 5e, 04103 Leipzig, Germany; 3 Centro Administrativo Parque Nacional Sierra Nevada, Carretera Antigua Sierra Nevada km 7, 18071 Pinos Genil, Granada, Spain; 4 Agencia de Medio Ambiente y Agua, Junta de Andalucía. C/ Johann G. Gutenberg 1, 41092 Sevilla, Spain; 5 Departamento Biología Animal, Biología Vegetal y Ecología, Universidad de Jaén, Campus Las Lagunillas, s.n., 23071 Jaén, Spain; 6 Departamento de Zoología y Antropología Física, Facultad de Biología, Universidad Complutense de Madrid, 28040 Madrid, Spain; 7 Fundación Oso Pardo, Calle San Luis 17, 4ºA, Santander, 39010 Spain

**Keywords:** Animal movement, Home range, Ibex, Integrated step selection analysis, Resource selection, Satellite-tracking

## Abstract

**Background:**

Space use by animals is determined by the interplay between movement and the environment, and is thus mediated by habitat selection, biotic interactions and intrinsic factors of moving individuals. These processes ultimately determine home range size, but their relative contributions and dynamic nature remain less explored. We investigated the role of habitat selection, movement unrelated to habitat selection and intrinsic factors related to sex in driving space use and home range size in Iberian ibex, *Capra pyrenaica*. We used GPS collars to track ibex across the year in two different geographical areas of Sierra Nevada, Spain, and measured habitat variables related to forage and roost availability.

**Results:**

By using integrated step selection analysis (iSSA), we show that habitat selection was important to explain space use by ibex. As a consequence, movement was constrained by habitat selection, as observed displacement rate was shorter than expected under null selection. Selection-independent movement, selection strength and resource availability were important drivers of seasonal home range size. Both displacement rate and directional persistence had a positive relationship with home range size while accounting for habitat selection, suggesting that individual characteristics and state may also affect home range size. Ibex living at higher altitudes, where resource availability shows stronger altitudinal gradients across the year, had larger home ranges. Home range size was larger in spring and autumn, when ibex ascend and descend back, and smaller in summer and winter, when resources are more stable. Therefore, home range size decreased with resource availability. Finally, males had larger home ranges than females, which might be explained by differences in body size and reproductive behaviour.

**Conclusions:**

Movement, selection strength, resource availability and intrinsic factors related to sex determined home range size of Iberian ibex. Our results highlight the need to integrate and account for process dependencies, here the interdependence of movement and habitat selection, to understand how animals use space. This study contributes to understand how movement links environmental and geographical space use and determines home range behaviour in large herbivores.

**Electronic supplementary material:**

The online version of this article (10.1186/s40462-017-0119-8) contains supplementary material, which is available to authorized users.

## Background

The extent of space animals use to live and reproduce, commonly known as home range, is considered a fundamental metric in animal ecology [1, 2]. Home range is defined by the interaction between animals and the environment, and its size is the direct result of movement driven by habitat selection and other external factors, biotic interactions, and intrinsic factors related to individual state and characteristics [2]. Although much progress has been done on understanding the processes underlying home range variation, integrative assessments are still lacking. One of the reasons is that movement driven by habitat selection is difficult to separate from movement driven by other factors (i.e. selection-independent movement) [3, 4].

Movement is the primary link between home range size and habitat/resource selection [5], although geographic and environmental space use have been usually addressed separately in the literature [6, 7]. Habitat selection affects home range size at different spatial scales: large-scale selection mediated by the availability and distribution of resources, landscape features and climatic conditions [8] (second-order selection; [9]); and fine-scale resource selection and use within home ranges (third-order selection; [9]). Home range size might also be affected by biotic interactions and intrinsic factors [2]. Known biotic interactions among animals include social interactions that lead to group dynamics, and territorial behaviour associated to reproduction strategies [10], whereas intrinsic factors include sex, age, and the internal state of animals [2].

Home range formation is thus the result of dynamic processes. Both the habitat and internal state of animals might change through time and cause home range size to vary. For example, seasonal variation might be determined by changes in selection according to variation in habitat preference within home ranges (third-order habitat selection) and/or by changes in resource availability and distribution across the landscape (second-order habitat selection) [11]. Broad-scale landscape dynamics might even trigger nomadic or migratory movements that allow animals to track changing resources over time [12, 13]. In addition to habitat selection, reproduction might affect movement during the mating and rutting seasons; as such, intrinsic factors such as sex might also lead home range size to vary among seasons (e.g. [14]).

In order to understand how these dynamic processes contribute to determine home range size, we performed an integrative analysis using movement data of a mountain ungulate (the Iberian ibex, *Capra pyrenaica*). Specifically, we investigated the relative contributions of habitat selection, selection-free movement and intrinsic factors (related to sex) to determine seasonal home range size. Although many of these factors have been reported to affect either habitat selection or movement in mountain ibex and large herbivores in general (see below), an explicit link between movement, habitat selection and home range size has never been made. Therefore, assessing the joint contribution of these factors will contribute to understand space use by animals. Large herbivores are excellent models to establish the link between primary productivity, selection and movement [15]. Accordingly, movement data might be related to environmental information collected through remote sensing at comparable spatial scales and resolutions [16].

The Iberian ibex is a gregarious species with virtually no natural predators in the study area, and thus territory defence and predation avoidance might not be as important as forage and roost selection for explaining space use. Indeed forage availability and the environmental factors that affect it, such as temperature, snow depth, rainfall and daylight, are key drivers of home range size in large herbivores [17, 18], including mountain ibex [19, 20]. Moreover, Iberian ibex live in mountainous areas with marked seasonality associated to altitudinal gradients, and track resource availability by performing progressive altitudinal movements [19]. Therefore, we hypothesised that selection strength and resource availability are key drivers of space use and home range size. We explored this hypothesis by using the selection coefficients derived from integrated Step Selection Analysis (iSSA), i.e. selection strength, as well as proxies of resource availability, including altitude, geographical location and environmental variables related to primary productivity, as predictors of home range size. Because ibex show altitudinal range shifts in response to resource availability, we broadened our definition of “home range” to include space covered during gradual altitudinal movements.

As an alternative or complementary process, we also considered the role of selection-independent movement in driving space use and home range size. For example, individual characteristics such as the animal’s internal state, territorial behaviour during the mating season or even personality might affect home range size. We expected that the displacement rate and directional persistence not affected by habitat selection would be positively related to home range size, assuming that larger scale landscape constraints are not as important as to restrict home range size. The recently developed integrated Step Selection Analysis allowed us to separate the effects of habitat selection from “selection-free” movement to explain space use.

Finally, differences between sexes in space use have been widely documented in ungulates [8]. Mountain ibex are sexually dimorphic, with males having a larger body size, which was shown to affect movement and selection behaviour [21]. Therefore, we hypothesised that sex is an important determinant of home range size, and predicted that females would have smaller home ranges due to restricted mobility resulting from parental care, especially during spring and summer when looking after newborns [22]; and/or smaller body size, which is a general predictor of home range size [23]. Restricted mobility can also lead females to select areas of higher habitat quality, allowing them to have smaller home ranges. On the other hand, there is a possibility of increased home ranges in females owing to higher energetic requirements derived from lactation [24].

## Methods

### Study area and species

The study was conducted in Sierra Nevada, mostly within the National Park (37°05′N, 3°28′W; SE Spain; Additional file 1: Figure S1). This park extends over 85,883 ha and is composed of mountains that rise over 3000 m a.s.l., ranging from 1700 to 3500 m. It is dominated by a continental Mediterranean climate with altitudinal gradients of temperature and rainfall. Rainfall is more frequent in spring and autumn, whereas summers are hot and dry and winters are cold with snowfall from November until April. The park has a highly diverse vegetation, with 2100 plant species (some endemic), structured in forests, shrubland and grassland along altitudinal gradients.

The Iberian ibex (*Capra pyrenaica*) is an endemic species of the Iberian Peninsula that inhabits mountainous systems [19]. This species live in social groups, but show spatial sexual segregation for most of the year, only coming together during the courtship (rutting) season, usually from October to December [25]. Kids are born in late spring, usually May. The Iberian ibex is a generalist herbivore, foraging as both a browser and grazer on a wide and varied diet that includes grass, shrubs and sometimes trees [19, 26]. Diet and foraging mode depend on resource availability [19]. Ibex can also perform altitudinal movements as to track seasonal resources that become available depending on climatic factors such as temperature and snow cover [19].

We conducted the study in two different geographical areas inhabited by different ibex population nuclei to control for possible effects of geographical idiosyncrasy, for example in the composition and density of resources. The areas differed mainly in altitude and vegetation cover, with the eastern nucleus being at lower altitudes and having a denser forest cover.

### Movement data

We equipped 22 Iberian ibex with GPS collars (Microsensory, Córdoba, Spain, and Vectronic Aerospace, Berlin, Germany) after capturing them by darting using an anaesthetizing mixture of xylazine (3 mg/Kg) and ketamine (3 mg/Kg). Their movement was monitored over a maximum of two years during 2005–2007 by obtaining positions every one to every four hours depending on the animal. Because some ibex died and some stopped transmitting data before completing at least a full season, we had a final sample of 18 animals (10 males and 8 females) living in two separated geographical areas of the mountains (9 in each of the two nuclei; Additional file 1: Figure S1). All the included ibex were tracked for multiple seasons. After removing the first five fixes and obvious relocation errors, we had a total of 2085–4639 fixes per animal. However, to homogenize the time lags between successive relocations across tracked animals, we subsampled the movement data to obtain relocations every four hours, rendering a total of 700–3230 fixes per animal over a temporal range of 206–576 days.

### Data analysis

We defined four different seasons according to the period of the year and the biology and life history of the Iberian ibex: spring (kidding season; April–June), summer (July–September), autumn (mating season; October–December) and winter (January–March). For each of the seasons, we first estimated for each of the 18 ibex habitat selection models that accounted for both movement and resource availability. These models allowed us to estimate selection and movement coefficients. Then, we explored to what extent selection-independent movement, selection strength, resource availability, and intrinsic factors (sex) determined seasonal home range size. All the specific analyses are described below.

#### Habitat selection models

Movement allows organisms to track the environment, and when estimating habitat selection, failure to account for the movement process may produce biased selection estimates [3]. A recent approach, termed integrated step selection analysis (iSSA) [4], builds on resource and step selection functions [27, 28] and can be used to model habitat selection while accounting for individual differences in movement behaviour. As such, this model can also be used to obtain estimates of selection-independent movement coefficients. We performed iSSA for each animal in each season to obtain individual, rather than population-level, estimates (as recommended in [4, 29]). iSSA simultaneously estimates movement and habitat selection parameters by comparing each used movement step with a set of conditioned available steps randomly sampled from an analytical distribution parameterised based on observed steps (*N* = 10 in this study). Movement steps were characterised by their length, i.e. the distance between the start-point and end-point of a given step, and direction, defined as the angular deviation (or turn angle) between successive steps. In particular, available step lengths were randomly sampled from a Gamma distribution fitted to observed step lengths of each animal in each season by maximum likelihood, and directions were randomly sampled from a uniform distribution of turn angles between successive steps.

Habitat covariate values were extracted for the end-point of each step and consisted of environmental variables related to foraging and roosting habitat: terrain slope (derived from a digital terrain model; www.juntadeandalucia.es/medioambiente/site/rediam), heat load (derived from the same digital terrain model) [30, 31], both with a spatial resolution of 10 m, and a primary productivity index (the Normalized Difference Vegetation Index, NDVI) obtained from satellite imagery (NASA product MOD13Q1; spatial resolution = 250 m, temporal resolution = 16 days). Because NDVI varies over time, each observed and control location was associated to the specific NDVI value corresponding to that location at the closest date. Heat load is an index of incident solar radiation that takes into account the orientation of the terrain slope, thus, depending on the latitude and season, it is associated to vegetation cover and snow depth. All habitat variables were centred and standardized and the respective quadratic effects included also as explanatory variables, as habitat selection might also show non-monotonic responses. Habitat variables were checked for collinearity by performing pair-wise correlation tests (correlation coefficients were all below 0.70; only in one case out of 76, we found a correlation lower than −0.70; Additional file 1: Table S1).

Each iSSA model included movement covariates, including step-length, its natural logarithm, and the angular deviation (cosine of the turn angle), as well as all habitat covariates mentioned above. Models were estimated using conditional logistic regression in the R package “survival” [32, 33]. The importance of habitat selection to explain space use was determined by comparing iSSA models containing only the movement covariates with models containing both movement and habitat covariates by means of the Akaike Information Criterion (AIC). In order to estimate mean step length (l_mean_) while accounting for habitat selection, we combined the estimated coefficients of the step-length with the parameter estimates of the Gamma distributions (used for sampling available step-lengths) as follows [4]:

$$ {l}_{mean}=\frac{k+\beta \ln (l)}{\theta^{-1}-{\beta}_l}, $$

where *k* and *θ* are the shape and scale of the observed Gamma distribution, respectively, and *β*_*l*_ and *β*_*ln(l)*_ are the iSSA coefficients for the observed step length and natural-log step length, respectively.

Because selection coefficients are not explicit about the range and values of used habitat, and might be dependent on other habitat covariates, we used the relative selection strength (RSS) [34] to show and interpret habitat selection results. RSS is defined as the probability of habitat use in one location over other locations. To obtain “population” (as defined by a group of interest) rather than individual RSS, we bootstrapped the mean RSS and calculated population 95% confidence intervals (see a similar approach in [35]).

#### Home range estimation

Home range size was estimated by calculating the area used by the different tracked animals through a bivariate normal utilization-kernel using the R package “adehabitatHR” [36]. We used the reference smoothing parameter (h_ref) [37] to estimate home ranges and the respective 90% and 50% contours (percentages chosen based on [38]), the latter representing an estimate of core areas. Home ranges were estimated for each animal in each season.

#### Determinants of home range size

Linear mixed-effects models (LMM) were used to test how home range size varies within and across seasons and what drives this variation. We hypothesised that season, selection-independent movement, selection strength, resource availability, and sex drive home range size. Selection-independent movement was characterised by the mean step length estimated from the iSSAs and the iSSA movement coefficient for the turn angle, which represents a measure of directional persistence (i.e. the concentration parameter of a von Mises distribution; [4]). Home range size was expected to increase with both step length (i.e. displacement rate) and directional persistence. iSSA selection coefficients were used as selection strength covariates. For resource availability, we used several proxies that included altitude and population nucleus as well as the mean and coefficient of variation (CV) of habitat variables related to forage availability (heat load and NDVI). It is worth noting that ibex were found to select a defined range of resource values, as consistently significant unimodal relationships were predicted by the iSSA models (see Results); therefore, higher CV (i.e. lower resource density) meant less resource availability.

The log-transformed home range size was used as the response variable, and the predictors included season (four-level factor), population nucleus (two-level factor), altitude, mean step length, directional persistence, slope selection (the linear and quadratic selection coefficients), heat load selection (linear and quadratic), NDVI selection (linear and quadratic), heat load availability (mean and CV), NDVI availability (mean and CV), and sex (two-level factor). Ibex identification was used as a random intercept effect. We performed model selection by comparing all models that included all possible predictor combinations by means of corrected AIC (AICc). Model estimation and selection were performed with the R packages “lme4” [39] and MuMIn [40], respectively. The relative importance of the different home range drivers was assessed by the difference in AICc when removing the target group of predictors.

## Results

### General space use patterns

**Fig. 1:**
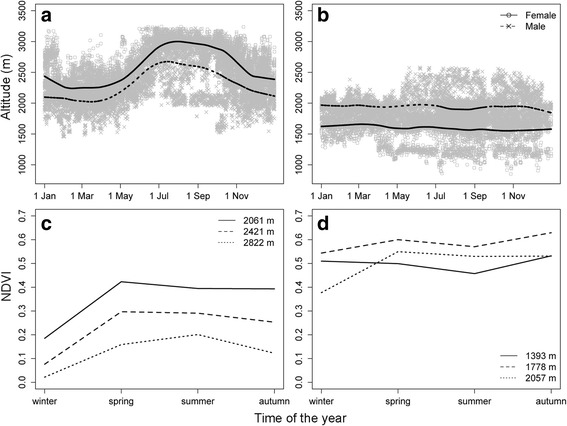
Altitude of tracked ibexes across the year for the western (**a**) and eastern (**b**) population nuclei, as well as seasonal variation of primary productivity (NDVI) at different altitudes in the western (**c**) and eastern (**d**) nuclei

The tracked ibex lived at high altitudes and had home range sizes of 0.39–33.17 km^2^ (90% kernel contour) and core areas of 0.08–10.79 km^2^ (50% kernel contour). Covered daily distances (net displacements) ranged from 332 to 3097 m, which were correlated with seasonal home range size (Pearson’s *r* = 0.90, *p* < 0.001, for the log-log correlation). Ibex performed altitudinal movements in the western nucleus, but not in the eastern nucleus, except for a few males that moved to higher altitudes during summer (Fig. 1a, b). In the western nucleus, ibex moved gradually to higher altitudes during spring, stayed at higher altitudes during summer, and descended during autumn, staying at lower altitudes during winter (Fig. 1a). Primary productivity (NDVI) followed the same seasonal pattern in the geographical areas where the western population lives. Vegetation cover increased from winter to spring, decreased from spring to summer at low and middle altitudes but increased at high altitudes, and decreased in autumn, except at lower altitudes were the NDVI increased again (Fig. 1c). In the eastern nucleus, primary productivity was more stable across the year (Fig. 1d), as this area is located at lower altitude and has a denser forest cover. We also observed sexual segregation in space, with females living at higher altitudes than males in the western nucleus, and the inverse pattern in the eastern nucleus (Fig. 1a, b), which suggests sexual differences in space use.

### Habitat selection

**Fig. 2:**
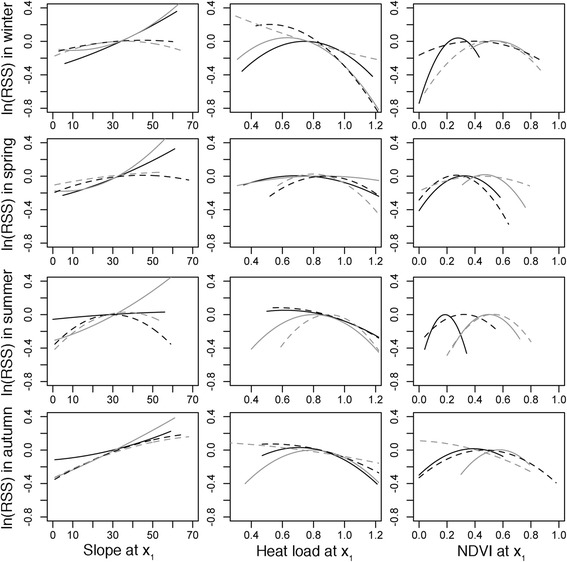
Relative selection strength (log-transformed RSS) for selecting location x_1_ over x_2_ (habitat value in x_2_ = 50%). The multiple panels correspond to all the combinations between season (rows) and habitat variable (columns). Continuous and dashed lines correspond to females and males, respectively; and dark and light lines correspond to the west and east nucleus, respectively. Note that only the mean RSS is shown to improve interpretation – see Additional file 1: Figure S2 for the figure with associated 95% confidence intervals

**Fig. 3:**
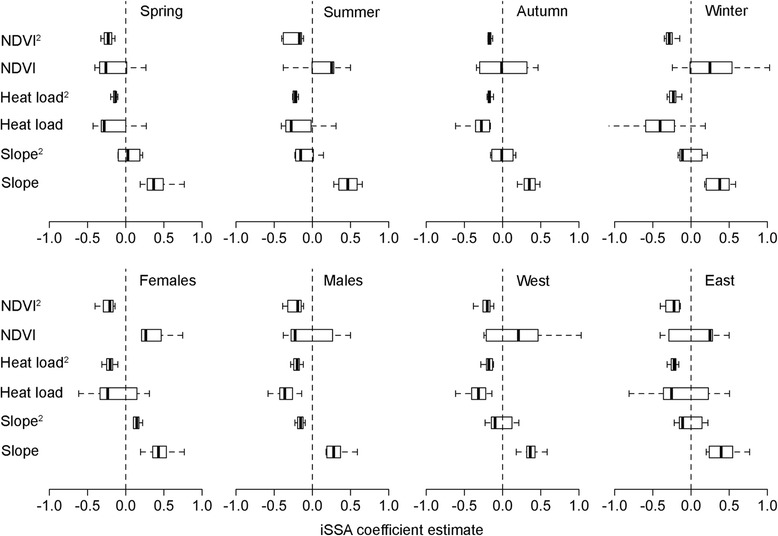
Boxplots of selection coefficients from the iSSA models pooled across seasons, sexes and population nuclei

In the iSSA models, the linear effects of slope and heat load were in general the most significant predictors of habitat selection (70 and 62% of the models, respectively): habitat selection increased linearly with increasing slopes (positive iSSA coefficients) and decreasing heat load (negative iSSA coefficients; Figs. 2 and 3). The quadratic effects of heat load and NDVI were also important in almost half of the cases (45 and 49% of the models, respectively), wherein selection increased up to optimum values of both heat load and NDVI but decreased again for higher values (negative iSSA coefficients corresponding to quadratic effects), indicating a unimodal relationship (Figs. 2 and 3). The quadratic effect of slope and the linear effects of heat load and NDVI were sometimes significant (28 and 33% of the models, respectively). Few seasonal trends in habitat selection were found, meaning that in general ibex selected similar habitat. Nevertheless, selection for both heat load and NDVI in winter tended to be stronger than in other seasons, whereas in the autumn (mating) season selection strength was overall reduced (Fig. 2). Differences between sexes and nuclei were larger (Fig. 2). Females tended to select steeper slopes than males, except in the autumn (mating) season, a pattern that was consistent between nuclei. Selection for heat load was more driven by sex, as males chose locations with lower heat load and females locations of intermediate heat load (unimodal relationship). However, in summer, lower heat load was selected by both males and females in the west (higher) nucleus than in the east nucleus (Fig. 2). For NDVI selection, differences between nuclei were larger, with ibex in the east nucleus selecting higher heat load and NDVI values (Fig. 3), though this was the result of local habitat availability rather than differential selection – selection coefficients were overall similar between nuclei (Fig. 3).

**Fig. 4:**
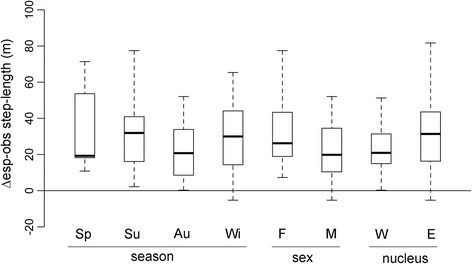
Difference between observed and estimated mean step-length pooled across seasons, sexes and population nuclei. Zero value represents the situation in which habitat selection does not influence movement behaviour and in turn space use

Space use by every ibex in every season was significantly determined by habitat selection (as estimated by comparing the AIC for iSSA models including and excluding habitat variables; difference in AIC > 2), except for one female ibex during spring. Estimated mean step length, once accounted for habitat selection, was almost always higher than the observed step length (Fig. 4), meaning that habitat selection constrained movement.

### Home range size

**Fig. 5:**
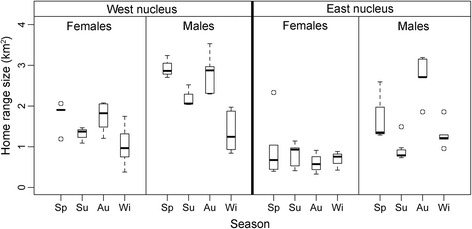
Home range size (90% contours) for the different combinations of season, sex and population nucleus

The most parsimonious model that explained the highest amount of variation in home range size (90% contour) included, by order of importance, selection-independent movement, selection strength, sex, season and resource availability (Table 1; only the results for the best model are shown). For the core area (50% contour) model, selection-free movement was again the most important predictor, followed by season, sex, selection strength, resource availability and nucleus. For both models, home range size increased with increasing displacement rate and directional persistence, as well as with increasing selection strength and resource dispersion (CV) (Table 1). Except for females in the eastern nucleus, which did not show seasonal variation, home range size was larger during spring and autumn and smaller during summer and winter (Fig. 5). In the west nucleus, where all ibex moved to higher altitudes, home ranges were generally larger than in the western nucleus (Fig. 5), where primary productivity is on average lower, though this was only significant for the core areas (50% home range contour). Males generally had larger home ranges than females (Fig. 5).

**Table 1:** Coefficients of the best home range size models (linear mixed models), for both the 90% and 50% contours. The AICc corresponds to a model in which the target group of predictors was removed, thus being a measure of its explanatory importance. NS, predictor not selected

	HR-90%			HR-50%		
Term	Estimate	S.E.	AICc	Estimate	S.E.	AICc
Intercept	−0.086	0.294		−0.269	0.241	
Season			90.141			58.017
Spring	−0.236	0.115		−0.200	0.082	
Summer	−0.402	0.121		−0.438	0.082	
Winter	−0.476	0.110		−0.461	0.079	
Sex			100.294			55.974
Male	0.740	0.123		0.582	0.097	
Nucleus			NS			38.772
East	NS	NS		−0.281	0.109	
Selection-free movement			129.895			69.538
Step-length	0.002	0.000		0.002	0.000	
Turn-angle	1.266	0.246		0.488	0.240	
Selection strength			101.627			44.634
Slope	0.827	0.194		0.504	0.141	
Slope2	2.306	0.544		1.261	0.422	
Heat-load2	1.645	0.373		0.780	0.273	
Resource availability			89.837			39.586
Heat load (CV)	4.252	1.637		4.070	1.390	
NDVI (CV)	0.457	0.163		NS	NS

Overall, qualitatively similar results were obtained for the 90% and 50% kernel contour of home ranges, and for each model a high proportion of home range size variation was explained (proportion of deviance explained by the marginal effects = 0.84 and 0.82, respectively). The only difference was that nucleus was selected only in the best core area (50% contour) model, and NDVI availability was selected only in the 90% home range model.

## Discussion

The patterns of space use by ibex in Sierra Nevada, namely home range size, varied across seasons and were determined by selection-independent movement patterns, resource selection strength and availability, and sex. Ibex that showed higher displacement rates and more directional persistence had, as expected, larger home ranges. This means that unmeasured habitat and landscape features, as well as (or) individual characteristics and motivations are important to explain individual variation in home range size. On the other hand, increased selection strength and less available or more scattered resources also led to larger home ranges. Finally, intrinsic characteristics related to sex also played a role, as males had larger home ranges than females. Reproduction behaviour and/or body size might explain the sex effect on home range size and sexual segregation.

The availability and distribution of preferred resources is a general driver of home range size of a wide diversity of animals [2, 41]. For example, herbivores show larger home ranges when forage is less available across the landscape [18]; and polar bears show larger home ranges when the distribution of their preferred prey (seals) is more unpredictable [17]. In Sierra Nevada, the altitudinal gradients in habitat availability during the spring season, e.g. forage quality and cooler temperature (lower heat load), progressively attract ibex to higher altitudes where fresh vegetation grows, which might explain the larger home range sizes. In autumn, vegetation at higher altitude becomes unavailable due to seasonal senescence and snowfall, leading ibex to descend back looking for fresh vegetation at lower altitudes, which again explains the larger home range sizes. On the contrary, during summer and winter, resources are more stable at higher and lower altitudes, respectively, and thus home range size can decrease accordingly. Differences between population nuclei are probably associated to different seasonal dynamics at different altitudes. At higher altitudes (in the western nucleus) snowfall is a major determinant of resource availability throughout the year, with resources becoming progressively available as snow cover retreats. However, at lower altitudes snowfall is not as intense and vegetation is denser (i.e. higher NDVI) and more stable across the year, which might provide constant forage. The differences in home range size between nuclei were explained by the resource availability effect, and this is probably the reason why the effect of nucleus and NDVI availability (CV) were interchanged between the 90% and 50% range contour models.

According to our expectations, home range size increased with selection-independent displacement rate and directional persistence. This might be due to individual differences in movement behaviour associated to the individuals’ internal state (physiological factors), morphology or even personality affecting, for example, activity, boldness and exploratory behaviour [2, 42]. Although we did not test for individual characteristics, the variation in habitat selection and use observed through the iSSA models (Figs. 2 and 3) suggest that individual differences explain some space use patterns. The higher variability in space use during winter (Fig. 2) is especially evident, and might indicate individual responses to winter conditions. Nevertheless, we cannot discard that unmeasured resources and landscape configuration, that could be implicitly driving the effect of selection-independent movement, also play an important role in home range behaviour. We also note that the relationship between displacement rate and home range size might depend on the temporal scale, for example with weekly or monthly home ranges.

As in other ungulate species, we also found evident sexual segregation in space, as indicated by differences in altitude across the entire year. Sexual segregation and consequent space use patterns have been widely discussed and seem to be common among ungulate species (e.g. [43, 44]), including the Iberian ibex [45]. Although we do not have a definitive explanation for this segregation, our results support both the reproductive strategy and forage selection hypotheses, which might be complementary rather than exclusive [44]. Accordingly, on one hand, females have to protect and feed their offspring to maximize their survival (as also observed in other ungulates) (e.g. [43]), and thus tended to choose steeper slopes, low to intermediate heat loads and more opened (less vegetated) areas. These habitats might provide more protection against predation. Although the ibex has no major predators in Sierra Nevada, small carnivores such as foxes and golden eagles can prey on young animals and foster innate anti-predatory behaviour (these ibex still have alarm calls in herds).

Females also showed smaller home ranges than males, which might partly be caused by restrictions to movement posed by raising their offspring, for example if the reduced mobility of young animals restricts the movement of females. On the other hand, males are less restricted by young ibex and predators, and might invest more time and space searching for food. This might explain their larger home ranges. Differences in home range size between sexes suggest that there might be a trade-off between foraging and reproductive strategies. According to the optimal foraging theory [46], the size of home ranges should be determined by the balance between the time and energy required to ultimately maximize fitness. Animals have to reproduce, consuming time and energy that could otherwise be used to increase forage efficiency. Because female ibex have the burden of raising offspring, a trade-off between foraging and reproductive strategies might underlie the observed smaller home range sizes. An alternative explanatory hypothesis is that males are bigger than females and thus have higher energetic demands that can be satisfied by having access to larger habitat patches [23].

Although we cannot discard the potential role of other movement drivers, such as territory defence behaviour, exploration of other types of resources, or predator avoidance in explaining observed space use patterns, these ibex are gregarious and have no natural predators (at least adults), thus these seem to be less important factors [19, 25, 26]. Further, we acknowledge that we dealt mainly with third-order habitat selection, i.e. selection within home range, and second-order selection related to resource distribution; however, habitat structure and landscape configuration at both broader and shorter spatial scales might influence space use and home range size by constraining movement at finer temporal scales and coarser spatial scales [8]. We highlight the importance of understanding how space use varies across time. Even finer temporal and spatial scales should provide further insight into habitat selection [47] and home range size [48]. For example, how does the importance of habitat selection and selection-free movement in explaining space use vary with temporal and spatial scale? Do these scaling relationships vary among individuals, species, and related traits? Such knowledge could provide an impartial tool to make comparisons across species and ecosystems that would contribute to delineate general mechanisms of home range behaviour. Still, we note the high proportion of variation explained by our home range size models, which suggest that a significant proportion of space use patterns might be explained by habitat selection and movement processes happening at the 4-h temporal scale and home-range spatial scale.

The Iberian ibex is currently undergoing a range expansion process throughout the Iberian Peninsula, both through natural dispersal and via reintroduction programmes [19, 49]. Although it has a conservation status of “least concern” by the IUCN due to current range expansion, two subspecies (*C. p. lusitanica* and *C. p. pyrenaica*) have already gone extinct (one in 2000) and another (*C. p. victoriae*) has a restricted distribution in the northwest Iberian Peninsula [19, 49]. Hence, understanding space use by the Iberian ibex may help to select introduction sites and predict colonisation patterns. The iSSA models allow generating maps of the expected utilisation distribution of animals across different landscapes, and may be a useful tool for management purposes [50].

## Conclusions

This study contributes to better understand the ecological determinants of home range behaviour and dynamics. Our results suggest that only an integrative assessment of both movement and habitat selection may allow us to understand home range size in large herbivores. Although space use should be studied at different temporal and spatial scales in the future, the dynamic nature of resource availability and individual responses to changing environmental conditions were important to explain movement behaviour and in turn space use patterns. This provides further insight into how movement ecology drives home range size and the dynamics of space use.

## Additional file

Additional file 1: Additonal tables and figures. (DOCX 1432 kb)
