# Supplementary material for: Linking seasonal home range size with habitat selection and movement in a mountain ungulate
Source: Mov Ecol. 2018 Jan 5;6:1. doi: 10.1186/s40462-017-0119-8 (PMC5755340; doi:10.1186/s40462-017-0119-8)

**Additional file 1**: additional tables and figures

Table S1. Pairwise correlations for the three habitat variables included in the iSSA models. The upper side of the matrix (above the identity diagonal) reports either the maximum or minimum correlation found among the 76 models, and the lower side (below the identity diagonal) reports either the 1^st^ percentile or the 99^th^ percentile of the correlations.

| Max |  |  |  |
| --- | --- | --- | --- |
|  | NDVI | Slope | Heat load |
| NDVI | 1 | 0.30 | 0.11 |
| Slope | 0.27 | 1 | 0.45 |
| Heat load | 0.09 | 0.45 | 1 |
|  |  |  |  |
| Min |  |  |  |
|  | NDVI | Slope | Heat load |
| NDVI | 1 | -0.63 | -0.45 |
| Slope | -0.53 | 1 | -0.75 |
| Heat load | -0.37 | -0.70 | 1 |

Table S2. Summary results for the 76 iSSA models. Predictor values are the model coefficients.

| Model ref. | Season | Year | Ibex ID | Step-length | Step-length (ln) | Turn angle (cos) | Slope | Slope^2^ | Heat load | Heat load^2^ | NDVI | NDVI^2^ | AIC |
| --- | --- | --- | --- | --- | --- | --- | --- | --- | --- | --- | --- | --- | --- |
| 1 | spring | 2006 | 1494 | 0.000 | 0.062 | 0.003 | 0.363 | 0.079 | -0.266 | -0.131 | -0.086 | -0.254 | 2080.084 |
| 2 | spring | 2007 | 1494 | 0.000 | 0.064 | 0.089 | 0.347 | 0.069 | -0.282 | -0.079 | -0.015 | -0.165 | 2284.015 |
| 3 | spring | 2006 | 1495 | 0.000 | 0.048 | 0.162 | 0.438 | 0.012 | -0.031 | -0.014 | 0.069 | -0.061 | 2071.634 |
| 4 | spring | 2007 | 1495 | 0.000 | -0.037 | 0.232 | 0.150 | -0.036 | 0.215 | -0.060 | 0.310 | -0.096 | 742.862 |
| 5 | spring | 2006 | 1497 | 0.000 | 0.031 | 0.026 | 0.331 | -0.012 | -0.034 | -0.113 | 0.195 | -0.200 | 1721.527 |
| 6 | spring | 2007 | 1498b | 0.000 | -0.015 | -0.040 | 0.190 | -0.015 | -0.002 | -0.096 | -0.239 | -0.203 | 1574.173 |
| 7 | spring | 2006 | 1502 | 0.000 | 0.025 | 0.141 | 0.132 | -0.054 | -0.036 | -0.132 | -0.116 | -0.325 | 2234.441 |
| 8 | spring | 2006 | 1503 | 0.000 | 0.014 | 0.355 | -0.065 | -0.093 | 0.049 | -0.098 | -0.158 | -0.071 | 2043.319 |
| 9 | spring | 2007 | 2037 | 0.000 | 0.032 | -0.114 | -0.337 | 0.022 | -0.428 | -0.039 | 0.265 | 0.011 | 1408.082 |
| 10 | spring | 2006 | 2038 | 0.000 | 0.026 | -0.134 | 0.369 | 0.042 | 0.125 | -0.160 | -0.279 | -0.063 | 1428.386 |
| 11 | spring | 2006 | 2040 | 0.000 | 0.074 | -0.613 | 0.430 | 0.029 | -0.092 | -0.104 | -0.404 | -0.047 | 1339.389 |
| 12 | spring | 2007 | 2041b | 0.001 | -0.037 | -0.490 | 0.108 | 0.086 | -0.336 | -0.122 | -0.152 | 0.092 | 1398.676 |
| 13 | spring | 2006 | 2042 | 0.000 | 0.271 | -0.444 | 0.575 | 0.221 | 0.268 | 0.028 | -0.256 | -0.262 | 1116.700 |
| 14 | spring | 2007 | 2042 | 0.000 | 0.204 | -0.651 | 0.546 | 0.149 | 0.269 | 0.055 | 0.020 | -0.302 | 947.165 |
| 15 | spring | 2007 | 2326a | 0.000 | 0.046 | -0.157 | 0.234 | -0.102 | -0.304 | -0.197 | 0.099 | -0.141 | 1504.185 |
| 16 | spring | 2007 | 2327 | 0.000 | 0.170 | -0.362 | 0.768 | 0.061 | 0.070 | -0.045 | -0.010 | -0.045 | 1268.522 |
| 17 | summer | 2006 | 1494 | 0.000 | 0.020 | 0.138 | 0.413 | -0.066 | 0.072 | -0.249 | -0.091 | -0.047 | 2057.268 |
| 18 | summer | 2006 | 1495 | 0.000 | -0.030 | 0.135 | -0.202 | -0.098 | -0.277 | 0.157 | -0.129 | -0.217 | 2436.355 |
| 19 | summer | 2006 | 1497 | 0.000 | 0.031 | 0.079 | -0.058 | 0.146 | -0.345 | -0.069 | 0.135 | -0.382 | 1790.799 |
| 20 | summer | 2005 | 1499 | 0.000 | 0.020 | 0.193 | -0.039 | -0.034 | 0.080 | -0.199 | 0.171 | -0.063 | 2295.992 |
| 21 | summer | 2005 | 1502 | 0.000 | 0.027 | 0.191 | 0.057 | -0.229 | -0.407 | 0.158 | 0.204 | -0.115 | 1970.739 |
| 22 | summer | 2006 | 1503 | 0.000 | 0.041 | 0.363 | -0.015 | -0.219 | -0.315 | -0.097 | -0.213 | -0.135 | 1866.009 |
| 23 | summer | 2006 | 2037 | 0.001 | 0.030 | -0.125 | 0.590 | -0.153 | -0.351 | -0.259 | 0.499 | -0.380 | 1144.215 |
| 24 | summer | 2007 | 2037 | 0.000 | 0.014 | -0.570 | 0.281 | -0.088 | -0.118 | -0.155 | 0.290 | -0.073 | 1473.695 |
| 25 | summer | 2006 | 2038 | 0.000 | 0.008 | -0.006 | 0.655 | -0.067 | 0.014 | -0.495 | -0.380 | -0.128 | 1347.063 |
| 26 | summer | 2006 | 2039a | 0.000 | 0.024 | -0.287 | -0.001 | -0.094 | 0.196 | -0.033 | 0.096 | -0.121 | 1411.315 |
| 27 | summer | 2006 | 2040 | 0.000 | 0.105 | -0.172 | 0.464 | 0.082 | -0.063 | -0.078 | 0.020 | -0.401 | 1250.516 |
| 28 | summer | 2006 | 2041b | 0.000 | 0.071 | -0.291 | 0.583 | 0.037 | -0.229 | -0.246 | -0.086 | -0.143 | 1275.912 |
| 29 | summer | 2007 | 2041b | 0.000 | 0.084 | -0.456 | 0.537 | -0.068 | -0.275 | -0.210 | -0.159 | -0.060 | 1487.177 |
| 30 | summer | 2006 | 2042 | 0.000 | 0.137 | -0.359 | 0.437 | 0.122 | 0.310 | -0.181 | 0.253 | -0.069 | 1043.041 |
| 31 | summer | 2006 | 2326a | 0.000 | 0.037 | -0.175 | 0.163 | -0.106 | -0.371 | -0.221 | 0.201 | -0.389 | 1316.042 |
| 32 | summer | 2006 | 2327 | 0.000 | -0.043 | -0.362 | 0.149 | 0.024 | 0.103 | -0.256 | 0.272 | -0.140 | 1171.129 |
| 33 | summer | 2007 | 2327 | 0.000 | 0.072 | -0.218 | 0.594 | 0.043 | 0.021 | -0.219 | 0.075 | -0.158 | 1484.430 |
| 34 | summer | 2006 | 2332 | 0.001 | -0.038 | -0.264 | -0.288 | -0.060 | 0.505 | 0.063 | -0.048 | -0.170 | 1559.479 |
| 35 | summer | 2007 | 2332 | 0.000 | 0.001 | -0.150 | -0.019 | -0.219 | -0.056 | -0.227 | 0.018 | -0.064 | 1491.769 |
| 36 | autumn | 2005 | 1493 | 0.000 | 0.072 | 0.055 | 0.370 | 0.174 | 0.061 | -0.171 | 0.465 | 0.018 | 1948.629 |
| 37 | autumn | 2005 | 1494 | 0.000 | 0.022 | 0.090 | -0.164 | 0.102 | -0.616 | -0.146 | -0.011 | 0.015 | 2302.899 |
| 38 | autumn | 2006 | 1494 | 0.000 | -0.012 | 0.311 | 0.067 | -0.082 | -0.354 | -0.014 | -0.010 | -0.178 | 1997.572 |
| 39 | autumn | 2005 | 1495 | 0.000 | 0.019 | 0.384 | 0.122 | -0.038 | -0.181 | -0.079 | 0.033 | -0.095 | 2107.110 |
| 40 | autumn | 2006 | 1495 | 0.000 | 0.042 | 0.288 | 0.194 | -0.040 | -0.170 | -0.101 | 0.219 | -0.178 | 2376.284 |
| 41 | autumn | 2005 | 1497 | 0.000 | 0.055 | 0.040 | 0.489 | 0.030 | -0.102 | -0.172 | 0.168 | -0.041 | 1811.926 |
| 42 | autumn | 2006 | 1497 | 0.000 | 0.035 | 0.256 | 0.312 | 0.030 | -0.042 | -0.081 | 0.061 | -0.152 | 2062.964 |
| 43 | autumn | 2006 | 1498b | 0.000 | 0.105 | 0.002 | 0.365 | 0.030 | -0.260 | 0.009 | -0.095 | -0.101 | 1438.732 |
| 44 | autumn | 2005 | 1499 | 0.000 | 0.041 | 0.170 | 0.342 | -0.124 | -0.294 | -0.119 | 0.000 | -0.129 | 2030.881 |
| 45 | autumn | 2005 | 1500 | 0.000 | 0.025 | 0.029 | 0.116 | -0.009 | 0.089 | 0.055 | 0.055 | -0.192 | 2101.704 |
| 46 | autumn | 2005 | 1502 | 0.000 | 0.076 | -0.129 | 0.410 | -0.013 | -0.159 | -0.094 | 0.044 | -0.257 | 2091.570 |
| 47 | autumn | 2005 | 1503 | 0.000 | 0.055 | 0.046 | 0.320 | -0.076 | -0.385 | -0.084 | -0.182 | -0.148 | 1944.311 |
| 48 | autumn | 2006 | 2037 | 0.000 | 0.091 | -0.318 | 0.218 | -0.009 | -0.389 | -0.013 | -0.244 | -0.073 | 1406.724 |
| 49 | autumn | 2006 | 2038 | 0.000 | 0.051 | -0.108 | 0.445 | -0.039 | -0.073 | -0.048 | -0.342 | -0.075 | 1616.626 |
| 50 | autumn | 2006 | 2039a | 0.000 | -0.039 | -0.203 | 0.278 | -0.157 | 0.009 | 0.050 | -0.300 | -0.025 | 1389.430 |
| 51 | autumn | 2006 | 2040 | 0.000 | 0.008 | -0.250 | 0.215 | 0.009 | -0.190 | -0.039 | -0.060 | -0.190 | 1388.112 |
| 52 | autumn | 2006 | 2041b | 0.001 | -0.001 | -0.186 | 0.288 | 0.113 | -0.313 | -0.175 | -0.122 | -0.081 | 1418.304 |
| 53 | autumn | 2006 | 2042 | 0.001 | 0.026 | -0.401 | 0.466 | 0.045 | 0.274 | -0.203 | 0.136 | -0.003 | 1239.175 |
| 54 | autumn | 2006 | 2326a | 0.000 | 0.001 | -0.216 | 0.359 | 0.001 | 0.037 | -0.033 | -0.075 | 0.053 | 1429.613 |
| 55 | autumn | 2006 | 2327 | 0.001 | 0.082 | -0.262 | 0.699 | -0.115 | 0.259 | -0.309 | 0.320 | -0.166 | 1148.913 |
| 56 | autumn | 2006 | 2332 | 0.000 | 0.030 | 0.055 | 0.117 | 0.015 | -0.300 | -0.034 | -0.154 | -0.061 | 1372.789 |
| 57 | winter | 2006 | 1493 | 0.001 | 0.157 | -0.179 | 0.542 | 0.213 | 0.188 | -0.251 | 0.195 | -0.246 | 1535.069 |
| 58 | winter | 2006 | 1494 | 0.001 | 0.030 | 0.024 | 0.153 | 0.035 | -0.378 | -0.271 | 1.030 | -0.284 | 1665.199 |
| 59 | winter | 2007 | 1494 | 0.001 | -0.011 | 0.267 | 0.073 | 0.003 | -0.500 | -0.120 | -0.217 | -0.350 | 2110.730 |
| 60 | winter | 2006 | 1495 | 0.000 | 0.022 | 0.146 | 0.394 | 0.022 | 0.122 | -0.198 | 0.215 | -0.304 | 1745.376 |
| 61 | winter | 2007 | 1495 | 0.000 | 0.041 | 0.029 | 0.499 | -0.105 | 0.034 | -0.200 | 0.127 | -0.090 | 2297.910 |
| 62 | winter | 2006 | 1497 | 0.001 | 0.000 | -0.071 | 0.361 | -0.043 | 0.133 | 0.002 | 0.747 | -0.201 | 1319.391 |
| 63 | winter | 2007 | 1497 | 0.000 | 0.040 | -0.030 | 0.530 | 0.025 | 0.147 | -0.211 | 0.208 | -0.070 | 1577.517 |
| 64 | winter | 2007 | 1498b | 0.000 | 0.116 | -0.276 | 0.584 | 0.003 | -0.140 | -0.048 | -0.244 | -0.066 | 1507.975 |
| 65 | winter | 2006 | 1499 | 0.001 | 0.025 | 0.021 | -0.575 | 0.033 | -1.138 | -0.143 | 0.083 | 0.007 | 1337.705 |
| 66 | winter | 2006 | 1500 | 0.000 | 0.053 | -0.061 | 0.002 | 0.072 | -0.580 | -0.285 | -0.020 | -0.120 | 1679.078 |
| 67 | winter | 2006 | 1502 | 0.001 | 0.012 | -0.015 | 0.137 | -0.074 | -0.404 | -0.117 | 0.101 | -0.181 | 1957.355 |
| 68 | winter | 2006 | 1503 | 0.000 | 0.137 | 0.068 | 0.180 | -0.169 | -0.807 | -0.181 | -0.156 | -0.111 | 1391.273 |
| 69 | winter | 2007 | 2037 | 0.001 | 0.079 | -0.203 | -0.305 | -0.119 | -0.812 | 0.100 | 0.327 | -0.254 | 1457.660 |
| 70 | winter | 2007 | 2038 | 0.000 | -0.008 | -0.247 | 0.239 | 0.027 | -0.062 | -0.085 | -0.041 | -0.147 | 1520.500 |
| 71 | winter | 2007 | 2039a | 0.000 | 0.026 | -0.094 | 0.105 | -0.145 | -0.001 | 0.010 | 0.078 | -0.322 | 1463.136 |
| 72 | winter | 2007 | 2041b | 0.000 | 0.141 | -0.334 | 0.200 | 0.094 | -0.607 | -0.118 | -0.232 | 0.003 | 1389.059 |
| 73 | winter | 2007 | 2042 | 0.000 | 0.082 | -0.401 | 0.398 | 0.145 | -0.221 | -0.313 | -0.050 | -0.118 | 1291.894 |
| 74 | winter | 2007 | 2326a | 0.000 | 0.032 | -0.217 | 0.248 | -0.033 | -0.212 | -0.065 | 0.248 | -0.338 | 1494.293 |
| 75 | winter | 2007 | 2327 | 0.000 | -0.003 | -0.204 | 0.392 | 0.015 | -0.236 | -0.282 | 0.076 | -0.090 | 1352.122 |
| 76 | winter | 2007 | 2332 | 0.000 | 0.007 | -0.178 | -0.094 | -0.048 | -0.448 | 0.134 | 0.176 | -0.049 | 1469.264 |

**Figure S1.** Elevation map of the study site and movement relocation data (dots). Dot colours correspond to different tracked ibexes.


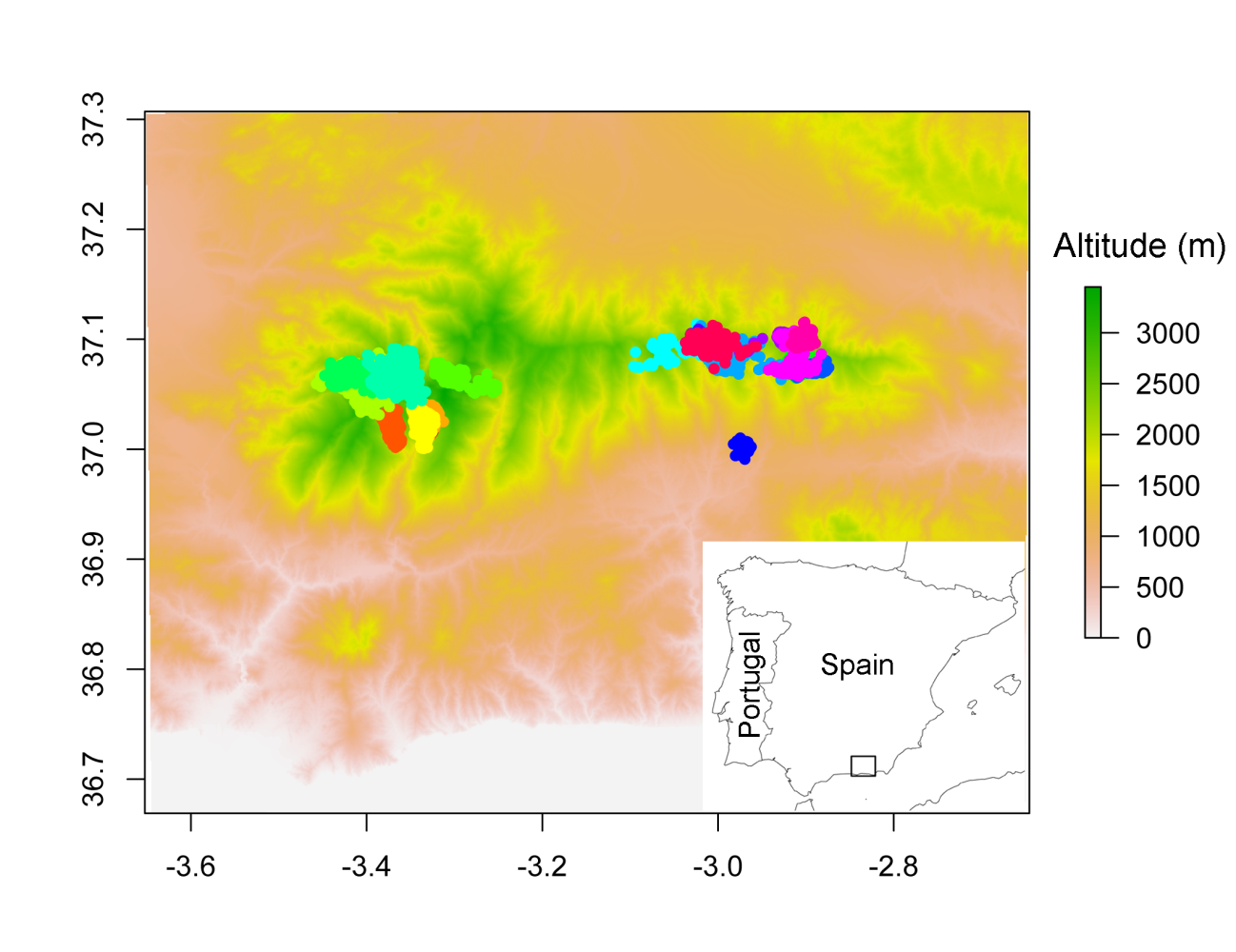


Figure S2. Relative selection strength (log-transfromed RSS) for selecting location x_1_ over x_2_ (habitat value in x_2_ = 50%). The multiple panels correspond to all the combinations between season (rows) and habitat variable (columns). Dashed lines correspond to 95% confidence intervals.


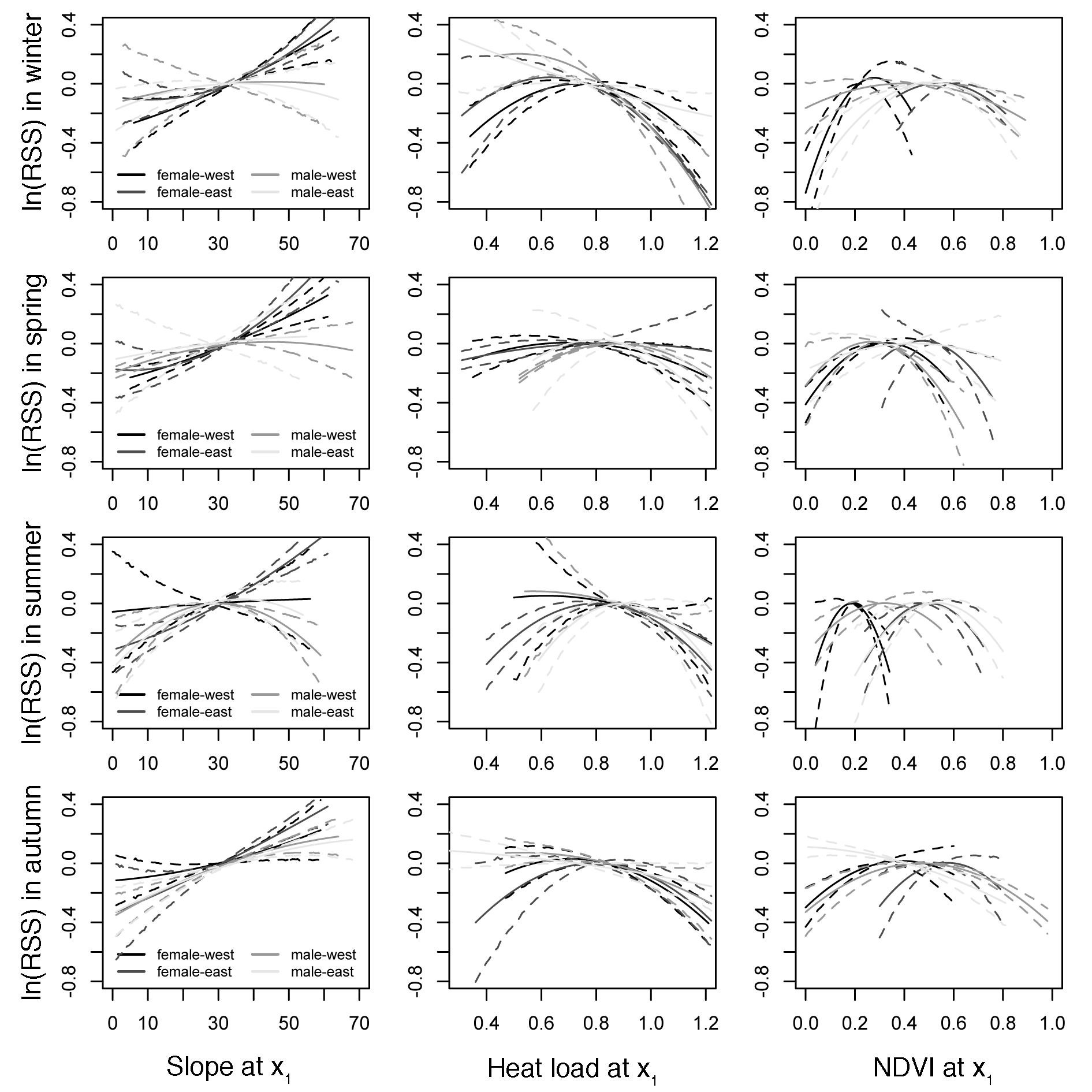

Supplement: Additional file 1: — Additonal tables and figures. (DOCX 1432 kb) [file 40462_2017_119_MOESM1_ESM.docx]
